# Goats favour personal over social information in an experimental foraging task

**DOI:** 10.7717/peerj.172

**Published:** 2013-09-24

**Authors:** Luigi Baciadonna, Alan G. McElligott, Elodie F. Briefer

**Affiliations:** Queen Mary University of London, Biological and Experimental Psychology, School of Biological and Chemical Sciences, London, UK

**Keywords:** Social learning, Patch assessment, Personal information, *Capra hircus*, Information use, Decision making

## Abstract

Animals can use their environments more efficiently by selecting particular sources of information (personal or social), according to specific situations. Group-living animals may benefit from gaining information based on the behaviour of other individuals. Indeed, social information is assumed to be faster and less costly to use than personal information, thus increasing foraging efficiency. However, when food sources change seasonally or are randomly distributed, individual information may become more reliable than social information. The aim of this study was to test the use of conflicting personal versus social information in goats (*Capra hircus*), in a foraging task. We found that goats relied more on personal than social information, when both types of information were available and in conflict. No effect of social rank was found on the occasions when goats followed other demonstrator goats. Goats are selective browsers/grazers and therefore relying on personal rather than social information could be the most efficient way to find patchily distributed resources in highly variable environments. Studies testing specific assumptions regarding the use of different sources of information can extend our understanding of decision making, including observed patterns of social learning.

## Introduction

Social learning is the process whereby animals acquire skills and knowledge by observing or interacting with other animals (Heyes, 1994). It has numerous advantages for animals because it constitutes a potential shortcut to acquire adaptive information, such as the location of valuable food or water resources, how to avoid predators, or how to move efficiently around an environment. Obtaining information by observing other individuals is generally less costly to acquire, in terms of the time and energy required to sample the environment, than personally acquired information (i.e., asocial learning) and allows flexible and fast acquisition of novel behaviours (Boyd & Richerson, 1985; Galef & Heyes, 2004; Galef & Laland, 2005; Thornton & Clutton-Brock, 2011). Social learning can give rise to culture, such as in humans (*Homo sapiens*) and some other species (Mesoudi, 2011; van de Waal, Borgeaud & Whiten, 2013). However, social information is not always as reliable as personal information and there might be circumstances in which it is better for animals to favour personal over social information (Boyd & Richerson, 1985; Galef, 2009; Kendal, Coolen & Laland, 2009). The choice of copying others could be influenced by specific characteristics of the situation, and of both the observer and the demonstrator (Rendell et al., 2011). Unfortunately, empirical studies investigating the strategies used by animals when copying others have been carried out on a limited number of species, especially in relation to how they consider personal versus social information (Laland, 2004; Laland & Galef, 2009).

Animals should copy a conspecific when a productive behaviour becomes unproductive, when acquiring or using personal information is too costly, or when there is uncertainty regarding the correct behavioural pattern to adopt in order to solve a problem or make a decision (“when strategies”; Galef, 1996; Kendal, Coolen & Laland, 2004). Generally, social learning is thought to be favoured at intermediate rates of environmental variability, because in highly variable environments, social information could be outdated or have no fitness benefit in the new environment (Boyd & Richerson, 1988). A common and supported assumption of these theoretical analyses is that individuals will be more likely to rely on social information if they lack personal information to guide their decisions, than if they possess reliable personal information (Laland, 2004; Galef, 2009; Kendal, Coolen & Laland, 2009).

Identity and other characteristics such as social rank, age, sex of both the demonstrators (“model-based biases”; “who-strategies”; e.g., Coussi-Korbel & Fragaszy, 1995; Duffy, Pike & Laland, 2009; van de Waal et al., 2010; Hopper et al., 2013), and the observers (“state-based biases”; e.g., Benskin et al., 2002; Webster & Laland, 2011), play important roles in the process of social learning (Laland, 2004). For example, according to the “copy-successful-individuals” strategy, animals should copy high-ranking individuals more often than low-ranking ones (Laland, 2004). However, few studies have attempted to test whether social status of the demonstrator (i.e., dominant vs subordinate) influences the likeliness of observers to learn socially (Nicol & Pope, 1999; Krueger & Flauger, 2007; Horner et al., 2010).

In this study, we investigated if goats use social information during a foraging task, and how they would trade-off personal or social information use when these two kinds of information were in conflict. Social learning has not been studied extensively in goats or other ungulates. The few studies on social learning in ungulates have explored only basic forms of social learning, such as “local enhancement” (when an individual’s attention is directed to a particular location by observing another individual; Hoppitt & Laland, 2008; e.g., Shrader et al., 2007; Krueger & Flauger, 2007; Held et al., 2000). Social learning from mothers is important in the development of foraging habits in young herbivores, including goats (Provenza, Pfister & Cheney, 1992; Glasser et al., 2009). Shrader et al. (2007) found that goats with no prior personal information use the only source of information available (conspecific behaviour) to locate high-quality patches. Thus, despite controversial evidence in some species (e.g., horses, *Equus caballus*, Krueger & Flauger, 2007), ungulates have the potential to acquire information through social learning, but it is not known if and how they trade off personal versus social information use during foraging.

Goats were probably the first livestock species to be domesticated (approximately 10,000 years ago, Zeder & Hesse, 2000). They live in large, complex social groups (Saunders et al., 2005; Stanley & Dunbar, 2013). Domestic goats follow the gaze of conspecifics towards outside objects/events (Kaminski, Call & Tomasello, 2006). They also use cues given by humans to locate hidden food, showing strong local and stimulus enhancement learning (Kaminski, Call & Tomasello, 2006). Recent research has shown that goat kids develop group-characteristic vocalisations through vocal learning (Briefer & McElligott, 2012), and goat mothers have long-term memory of their kids’ calls (up to 13 months after separation; Briefer, de la Torre & McElligott, 2012). Goats are selective browsers/grazers that thrive in different, harsh environments that also require them to use a wide range of resources (Coblentz, 1978; Goetsch et al., 2010; Duncan & Young, 2002). These social and ecological factors suggest that they could benefit from social learning.

This study investigated the strategy adopted by goats in a foraging task, when only social information (and no personal information) was available, as well as the use of personal and social information, when these two kinds of information were available and in conflict. This experimental design allowed us to test “when” and “who” strategies (Laland, 2004; Kendal, Coolen & Laland, 2004). More specifically, we tested (1) whether goats would use social information provided by the demonstrator when no prior personal information about where the food was located was available (i.e., without any training), and (2) whether the goats would use social or personal information when prior personal information about food location that contradicted social information was available (i.e., after training). These conditions were tested in combination with the “who” strategy of “copying successful-individuals” (i.e., higher-ranking individuals), in order to determine whether the use of social information was influenced by the demonstrator’s social rank. This is based on the assumption that higher-ranking individuals might be considered more successful by conspecifics (Nicol & Pope, 1999).

We based our predictions on previous research showing that goats alter their feeding behaviour according to both the status of group members and the sources of information available to locate high quality food patches (Barroso, Alados & Boza, 2000; Shrader et al., 2007). Our predictions were also based on theoretical models and experimental evidence for the use of social versus personal information in other species (Boyd & Richerson, 1988; Giraldeau, Valone & Templeton, 2002; Kendal, Giraldeau & Laland, 2009; Galef, 2009). Accordingly, we expected goats to be more prone to use personal information when that information was available (condition 2), and to copy others in the absence of personal information (condition 1). We also expected that the use (or not) of social information would be influenced by the rank of demonstrators. Goats could be more willing to copy higher-ranking individuals, if they consider them as successful or if they pay more attention to them (Nicol & Pope, 1999; Galef & Laland, 2005). Alternatively, observers could copy lower-ranking more than higher-ranking individuals, if fear of aggression prevents them from watching or learning from dominant demonstrators (Nicol & Pope, 1999). The study of personal and social information use in ungulates can provide important information about how animals in general gather information and make important decisions.

## Materials & Methods

### Subjects and management conditions

The experiments were conducted from May to November 2012, and involved 28 adult horned and dehorned female and male goats of various breeds and ages (Table 1). The animals were housed at an animal sanctuary (Buttercups Sanctuary for Goats, http://www.buttercups.org.uk) in Kent, UK. Fourteen goats were used as demonstrators and 14 as observers (test subjects). The same observers and demonstrators were used for the two conditions of our experiment (i.e., without and with prior personal information). All had lived at the sanctuary for at least eight months. All the goats were released into a large field during the day and were therefore familiar with each other. At night, they were kept indoors in individual or shared pens (2 or 3 goats, average size = 3.5 m^2^) with straw bedding, within a larger stable complex.

**Table 1:** Characteristics of the goats used in the experiment. Breed, sex, age, presence of horns, Clutton-Brock Index (CBI) of dominance and actual dominance rank of the goats used as observers and demonstrators (low-ranking, “LR”; highranking, “HR”). The description of the observer-demonstrator pairs formed is also shown.

Subject	Breed	Sex	Age	Horned	Demonstrator/ Observer	CBI	Dominance rank	Paired with
1	British Boer	M	5	No	LR Demonstrator	0.008	69	8, 9
2	Mixed breed	M	10	No	LR Demonstrator	0.011	67	10, 11
3	Golden Guernsey	M	11	No	LR Demonstrator	0.012	66	12, 13
4	British Saanen	F	2	No	LR Demonstrator	0.012	66	14, 15
5	Golden Guernsey	M	7	No	LR Demonstrator	0.036	63	16, 17
6	Golden Guernsey	F	NA	No	LR Demonstrator	0.057	60	18, 19
7	Mixed breed	F	10	No	LR Demonstrator	0.063	59	20, 21
8	Golden Guernsey	F	12	No	Observer	0.064	58	1, 22
9	British Saanen	F	8	No	Observer	0.072	56	1, 22
10	Mixed breed	F	10	No	Observer	0.091	55	2, 23
11	Pygmy	M	4	Yes	Observer	0.120	53	2, 23
12	Anglo-Nubian	F	12	No	Observer	0.160	49	3, 24
13	Pygmy	M	5	Yes	Observer	0.214	48	3, 24
14	British Toggenburg	M	8	Yes	Observer	0.243	47	4, 25
15	British Saanen	M	10	No	Observer	0.316	45	4, 25
16	Pygmy	M	15	Yes	Observer	0.370	44	5, 26
17	Pygmy	M	12	Yes	Observer	0.740	37	5, 26
18	Pygmy	F	9	Yes	Observer	0.915	32	6, 27
19	British Toggenburg	F	14	Yes	Observer	1.235	29	6, 27
20	Pygmy	M	9	Yes	Observer	1.807	23	7, 28
21	Pygmy	M	11	Yes	Observer	2.037	21	7, 28
22	British Alpine	F	8	Yes	HR Demonstrator	2.158	20	8, 9
23	Pygmy	M	13	Yes	HR Demonstrator	2.292	18	10, 11
24	British Toggenburg	M	11	Yes	HR Demonstrator	3.333	15	12, 13
25	British Alpine	F	6	Yes	HR Demonstrator	3.625	13	14, 15
26	British Toggenburg	F	8	Yes	HR Demonstrator	5.800	9	16, 17
27	Pygmy	M	15	Yes	HR Demonstrator	10.539	7	18, 19
28	British Toggenburg	M	8	Yes	HR Demonstrator	193.00	2	20, 21

Routine care of the animals was provided by sanctuary employees and volunteers. Goats had ad libitum access to hay, grass (during the day) and water, and were also fed with a commercial concentrate in quantities according to their state and age. Every stable was cleaned on a daily basis. All goats were inspected each day by the sanctuary employees and volunteers, were checked regularly by a vet and given medication when appropriate. Animal care and all experimental procedures were in accordance with the guidelines of The Association for the Study of Animal Behaviour. The research plan was reviewed by the UK Government Home Office inspector for Queen Mary, University of London.

### Observers and demonstrators

Each observer was paired with two demonstrators according to their respective dominance ranks (Table 1). To assess the dominance hierarchy, the entire herd (150 goats) was observed from March 2011 to April 2012 on a regular basis (two times per week). All events recording of agonistic interactions, including fights, displacements, threats, aggressions (head butts) and retreats were carried out. The outcomes of agonistic interactions resulting in a clear “winner” and “loser” (the winner wins the fight, displaces, threats or aggresses the loser that, as a consequence, retreats; *N* = 579 interactions involving 98 goats) were used to calculate the ranks of all goats that interacted with at least 5% of other goats. Because goat herds typically have linear hierarchies (Barroso, Alados & Boza, 2000), we calculated dominance ranks using the Clutton-Brock Index (Clutton-Brock et al., 1979; Bang et al., 2010) as follows: Clutton-Brock Index (CBI) = (B + b + 1)/(L + l + 1), where B is the number of goats defeated by the focal goat (“losers”), b is the number of goats (excluding the focal goat) defeated by the losers, L is the number of goats that defeated the focal goat (“winners”) and l is the number of goats that defeated the winners. The goat with the highest index was assigned the rank of 1, and all the other goats were ranked accordingly. In total, 72 goats were ranked.

The goats at the top of the hierarchy (ranks 2–20) were used as higher-ranking demonstrators and the ones towards the bottom of the hierarchy (ranks 59–69) were used as lower-ranking demonstrators (Table 1). The goats with intermediate ranks between the lower-ranking and higher-ranking demonstrators (ranks 21–58) were used as test subjects (observers). Then, each observer was paired with a higher-ranking demonstrator and a lower-ranking demonstrator that did not share its pen at night (Table 1). The rank difference between the observers and the higher-ranking demonstrators was 30.6 ± 1.7 ranks (range = 19–38), and it was 21.5 ± 2.4 ranks (range = 11–38) between the observers and the lower-ranking demonstrators. These large differences in ranks between observers and demonstrators, associated with the existence of clearly established, stable and linear hierarchies in goat herds (Barroso, Alados & Boza, 2000), ensured that our classification of demonstrators as higher- or lower-ranking than observers accurately depicted rank relationships. Each observer was tested twice for each of the two tested conditions; once with the higher-ranking demonstrator and once with the lower-ranking demonstrator in a random order (i.e., in each condition, half of the observers started with the lower- ranking demonstrator and the other half with the higher-ranking demonstrator).

### Experimental apparatus

**Figure 1:**
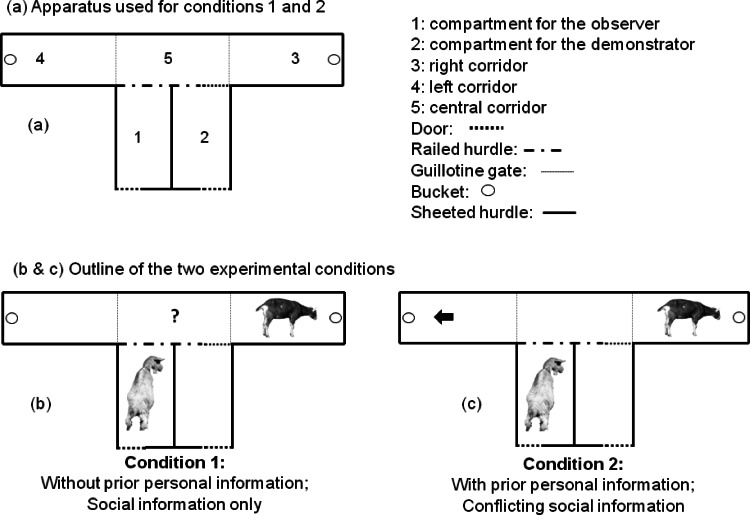
Experimental apparatus (A) and outline (B and C) for the two experimental conditions. Condition 1 tested whether observer goats without prior personal information about food location would copy the demonstrator (i.e., use social information to locate food). Condition 2 tested whether observers with prior information about food location (i.e., that had learned to find food on one side of the experimental apparatus, indicated by the black arrow) would copy the demonstrator and choose the opposite side than the one where they had been trained to go (i.e., use social information in conflict with their personal information). For each condition, observers were tested twice, once with a higher-ranking demonstrator, and once with a lower-ranking demonstrator.

The experimental apparatus was set up in an open field, which was part of the normal daytime range of the goats. It consisted of two compartments and three corridors (Fig. 1A). Compartments 1 and 2 (2 m × 1 m each) were used for the observer and demonstrator. Compartment 2 was connected to corridor 5 (central) by a door in railed hurdle. The corridor 5 allowed the animal to make a choice between corridors 3 and 4 (left and right, 2 m × 1 m). At the beginning of each of the left and right corridor (3 and 4), there was a guillotine gate, which was open or closed according to the different phases of the experiment. A blue bucket containing the food was placed at the end of each corridor (two blue buckets in total, empty or not, depending on the corridor and trial) and covered with an 8 mm thick plastic lid, in order to prevent any olfactory cues indicating the location of the food reward (Briefer & McElligott, 2013). The blue buckets at the end of each corridor, were present and visible during all phases of the experiment. Compartments 1 and 2 had three sides covered with opaque iron sheeted hurdles, and one side with a rail, which allowed the observers to see clearly the demonstrators entering corridor 5, and going to feed in the bucket at the end of corridors 3 and 4.

### Training of the demonstrators

The demonstrators were trained to choose one specific corridor, reinforcing this choice with the presence of food only in the bucket at the end of the targeted corridor. Four of the higher-ranking demonstrators were trained to go to the left corridor and three to the right. Similarly, four of the lower-ranking demonstrators were trained to go to the left corridor and three to the right (corridors 3 and 4 in Fig. 1A). During the training procedure, the individual was brought to compartment 2 for 90 s. At the end of the 90 s, the gate to access the central corridor 5 was opened to allow the animals to reach the food. Initially, they received two training sessions of four trials each. In order to facilitate learning, during the first training session, only the guillotine gate to access the correct corridors 3 or 4 (rewarded or unrewarded, alternatively) was open. During the second training session as well as during all the tests (condition 1 and 2 described below), access to both compartments was allowed (i.e., both guillotine gate were opened). The two training sessions were carried out during two consecutive days. Afterwards, demonstrators received one reminder trial before each test condition. During these reminder trials, the percentage of correct choices (i.e., consistent with the training) obtained was 100% for the two conditions.

## Experimental Procedure

### Condition 1: goats without prior personal information

The aim of the first experiment was to test whether the observers, which had not received any training on the position of food (“without personal information”), would base their decision on the choice of the demonstrators (i.e., use social information; Fig. 1B). We also tested whether this decision was influenced by the dominance rank of the demonstrators. The test was carried out during the week following the initial training of the demonstrators. The observers were tested without any prior training. During each test, the observer was placed in compartment 1 and the demonstrator in compartment 2 for 90 s. At the end of the 90 s, the experimenter attracted the attention of the observer towards the side with the rail, to ensure that it would be able to see clearly the demonstrator eating, and moved away to open the door of compartment 2, allowing the demonstrator to access the central corridor (corridor 5 in Fig. 1A) and to access the food in the corridor where it had been trained to go (corridors 3 or 4 in Fig. 1A). The demonstrator was then moved away from the apparatus, and the observer was brought from compartment 1 to compartment 2 and stayed there for 90 s. At the end of the 90 s, the door was opened to allow the access to the corridor 5. The choice of the corridors 3 or 4, defined as at least two forelegs positioned beyond the line of the gate, was recorded. The observers were never rewarded at any stage of the tests, in order to avoid any association between the reward and the decision to copy or not the demonstrators.

### Condition 2: prior personal information in conflict with social information

The aim of the second experiment was to test whether the observer goats, which had received training about the position of food (“with personal information”), would prefer to base their decision on the demonstrator’s behaviour or on their personal information (Fig. 1C). We also tested whether this decision was influenced by the dominance rank of the demonstrators. This condition consisted of two steps: (a) training each observer to enter the corridor opposite the one where its paired high- and low-ranking demonstrators had been trained to go; (b) testing the observers.

(a) Training of the observers: observers were trained following a similar procedure as for the demonstrator (see *Training of the demonstrators* above). The observers received one session of six trials with only one corridor open to facilitate learning, followed by one session of four trials during the following day, in which both corridors 3 and 4 were open. Before the test, one reminder trial was provided (i.e., without demonstrator). The percentage of correct choice (i.e., consistent with the training) for the observers during this reminder trial was of 92.85%. During this phase, the demonstrators received two reminder sessions about the training previously received (one trial each during two consecutive days). (b) Testing of the observers: test phase followed the same procedure as in condition 1.

### Statistical analyses

We used generalized linear mixed models (GLMM) fit by the Laplace approximation and with restricted estimate maximum likelihood (lmer function in R; Bates, Maechler & Bolker, 2011), to test whether the choices of the goats to go to the same side as the demonstrator or not differed between the two conditions and was influenced by the dominance rank and sex of the demonstrator. The choice of the goats was included as a response variable (coded as: went to the same side as the demonstrators = 1; went to the non-demonstrator side = 0). The explanatory variables were (1) the side where the demonstrator had been trained (right or left) to control for potential side biases; and (2) the condition (condition 1 or 2), to test for the effect of the availability of personal information on social information use (no personal information in condition 1 versus prior personal information in condition 2). To test for the effect of the demonstrator’s sex and rank on the use of social information, we also included as explanatory variables (3) the sex of the demonstrator and (4) the dominance rank of the demonstrator (higher or lower ranking). The identities of observers and demonstrators were included as random effects to account for repeated measurements of the same individuals within and between experiments. We fitted this model with a binomial family distribution and logit link. Furthermore a Chi-square test was used in each condition to investigate whether the data were different from an even distribution.

## Results

### Condition 1: goats without prior personal information

The proportion of goats that went to the same side as the higher-ranking demonstrator was 57.14% (8/14 subjects). These results did not differ from an even distribution (Chi-square test: ${\chi }_{1}^{2}=0.29$, *P* = 0.59). The proportion of goats that went to the same side as the lower-ranking demonstrator was 42.85% (6/14 subjects). These results also did not differ from an even distribution (Chi-square test: ${\chi }_{1}^{2}=0.29$, *P* = 0.59). Ten out of 14 observers that chose one corridor during the first trial chose the opposite corridor during the second trial. Among these 10 observers, seven chose the opposite side from the first demonstrator during the first trial and then went to the same side as the other demonstrator during the second trial. The remaining three observers chose the same side as the first demonstrator during the first trial and then went to the opposite side from the other demonstrator during the second trial. Of the four observers that went twice to the same side, two went to the same side as both demonstrators during the two trials, and two went to the other side on two occasions. Thus, goats did not show a preference for the side of the demonstrator.

### Condition 2: prior personal information in conflict with social information

None of the goats went to the same side as the higher-ranking demonstrator (0/14 observers). The proportion of goats that went to the same side as the lower-ranking demonstrators was 14.28% (2/14 observers), compared to 85.71% of the subjects (12/14 subjects) that went to the other side. These results differed from an even distribution (Chi-square test: ${\chi }_{1}^{2}=7.14$, *P* = 0.008). Goats thus used their personal information more than social information.

### Rank effect and comparison between the two experimental conditions

Over the two conditions, the dominance rank of the demonstrators did not affect the side where the observers went (*N* = 7 observers went to the same side and 21 did not go to the same side as the higher-ranking demonstrator; *N* = 9 observers went to the same side and 19 did not go to the same side as the lower-ranking demonstrator; GLMM: *Z* = 0.68; *N* = 56 tests, 14 observers; *P* = 0.50). Similarly, the sex of the demonstrator did not affect the side where the observers went (*N* = 8 observers went to the same side and 16 did not go to the same side as female demonstrators; *N* = 8 observers went to the same side and 24 did not go to the same side as male demonstrators; GLMM: *Z* = −0.60; *N* = 56 tests, 14 observers; *P* = 0.55). However, the choices of the observers differed depending on the experimental condition (1 and 2; GLMM: *Z* = −3.15; *N* = 56 tests, 14 observers; *P* = 0.002). To summarize, the goats did not show a preference for the side of the demonstrator when they did not possess personal information about food location, and they favoured personal information when they were trained to find food in one specific location.

## Discussion

Individuals are assumed to benefit from using social information, because by doing so they gain from acquiring adaptive information in a less costly manner than when they use individual learning (Kendal, Coolen & Laland, 2009). Although trade-offs in the use of social and personal information have been tested in fish, rats and birds (Laland, 2004; Galef, 2009; Lefebvre & Boogert, 2010), studies using other species are rare. Our primary aim was to investigate whether goats use social information and how they trade off personal and social information use during a foraging task. Our results show that goats did not show a preference for the side of the demonstrator in the foraging task. Most goats (10/14) changed sides between the first and second trial, suggesting that they used an exploratory strategy when they did not have any personal information (condition 1). When both personal and social information were available but in conflict, goats relied more on personal than social information (condition 2). Our secondary aim was to test whether the dominance status of the demonstrator could influence the likelihood of social information being used. Our results show that when goats went to the same side as the demonstrator, this choice was not influenced by social dominance. We propose that social information might not be preferentially used by goats, because individual information is likely to be optimal for finding food in this species. The study of adaptive use of social and asocial information has the potential to increase our understanding of how animals interact with the social and physical environments in which they live (Rendell et al., 2011; Thornton & Malapert, 2009).

### Condition 1 and 2: goats favour the use of personal over social information

The results found in condition 1 (i.e., not preferentially copying the demonstrator when no prior personal information is available) could be attributed to different causes. First of all, our results could result from the nature of the task used in our experiments. In our test, observer goats watched the demonstrator eat and were then tested after being moved to another compartment, followed by an interval of 90 s. In order to copy the demonstrator, goats had to learn by observation and then go to the same side, once the demonstrator had been removed from the setup (“delayed local enhancement”; Hoppitt & Laland, 2008). This task could be more complex for goats than simply joining group members that are eating at a given patch (local enhancement), due to an aggregation tendency (Shrader et al., 2007). Using an insect model, learning food location by observation has recently been shown to arise through simple association between the presence of conspecifics and a food reward (Dawson et al., 2013). In our experiment, goats had not been previously rewarded for copying conspecifics. Our results could thus be explained by the fact that goats had not yet formed an association between the action of copying the demonstrator and presence of food. Further experiments, in which goats are trained to associate demonstrators with food, could be carried out to test whether they can develop (through second-order conditioning, e.g., Dawson et al., 2013), this ability or motivation to learn by observation. Yet, our results suggest that this is not an ability that is naturally found in goats, nor in other ungulates, as similar results have been found with horses (e.g., Clarke et al., 1996; Lindberg, Kelland & Nicol, 1999).

Goats may have had a tendency to avoid the side that had potentially been food depleted by the demonstrator. However, 7 of the 9 goats that in the first trial of the first condition went to the opposite side as the demonstrator, then went to the same side as the second demonstrator in the other trial. This pattern is more indicative of an exploratory strategy than a simple tendency to avoid a food-depleted area.

Our results for condition 2 are consistent with theoretical models and experimental evidence in fish *(Pungitius pungitius)*, rats (*Rattus norvegicus*), and dogs (*Canis familiaris*). Individuals prefer to use prior and reliable personal information rather than social information for foraging decisions in stable environments (Pongrácz et al., 2003; van Bergen, Coolen & Laland, 2004; Kendal, Coolen & Laland, 2004; Galef, 2009).

According to Kendal, Coolen & Laland (2009), species that use complex foraging skills (e.g., cooperative hunting, tool use) could rely on social information more than individual learning. Herbivores are known to socially learn how to choose food items and avoid toxic foods from a very early age (Provenza, Pfister & Cheney, 1992; Glasser et al., 2009). They also monitor the eating behaviour of group members and minimize the risk of predation, by choosing food patches closer to groups (Shrader et al., 2008). However, they might not have evolved to be dependent on social learning processes other than local enhancement to find food patches. Social learning is likely to evolve when environments vary only at an intermediate level, either spatially or temporally (Boyd & Richerson, 1988). Therefore, the potentially patchily distributed resources (Goetsch et al., 2010) with which wild or feral goats have to cope could make social learning other than local enhancement (e.g., delayed local enhancement) less adaptive (Boyd & Richerson, 1988). The use of social learning strategies could become less likely if demonstrators and observers interact with different environments.

An absence of social information use can be due to the potential disadvantages of using acquired social information. Giraldeau, Valone & Templeton (2002) proposed two of these possible disadvantages. The first one corresponds to cognitive or physical restrictions to acquire two different types of information (personal and social) simultaneously. Second, the risk of informational cascades resulting in sub-optimal behaviour, which can arise because copying is based on very little information. Asocial learning is believed to be more often reliable than social learning, because it requires individuals to interact directly with their environment, which results in accurate, current information and is not prone to copying errors (Galef, 2009). Social learning could therefore be a last resort when asocial learning has failed (Kendal, Coolen & Laland, 2009).

### Influence of rank on the use of social information

Understanding the characteristics of the demonstrator that are salient for the observer according to the species they belong to, may offer new insights into the use of the “who” strategies (Nicol & Pope, 1999; Galef, 2009; van de Waal et al., 2010). Overall, we did not find a link between the use of social information and the social status of the demonstrators (dominant or subordinate). This could suggest that dominant goats are not better at foraging than subordinates and their foraging decisions are thus not copied by other individuals.

## Conclusion

To conclude, our study suggests that goats favour the use of personal over social information when foraging. The preference for personal over social information use in our study could be due to the specific characteristics of the task (e.g., delay between observation of the demonstrator and food choice) and the resulting motivation of the goats to use social information. Our study constitutes a first step towards an understanding of the conditions under which domesticated goats use social learning in foraging tasks.
